# Malignancy and immune disorders in patients with hereditary angioedema

**DOI:** 10.1186/s13223-021-00621-7

**Published:** 2021-12-19

**Authors:** Peter Stepaniuk, Amin Kanani

**Affiliations:** Division of Allergy and Immunology, Department of Medicine, University of British Columbia, 905-750 Broadway W, Vancouver, BC V5Z 1H8 Canada

**Keywords:** Hereditary angioedema, Malignancy, Cancer, Complement, Immune disorder, Autoimmune

## Abstract

**Background:**

Hereditary angioedema (HAE) is an inherited condition manifesting as recurrent angioedema episodes which is caused by deficiency or dysfunction of C1 inhibitor. Although complement dysregulation has historically been shown to be associated with various malignancy and immune disorders, it is currently not known if HAE patients are at an increased risk of developing malignancy or autoimmune conditions.

**Case presentation:**

We reviewed the charts of 49 HAE patients and identified 6 patients who had a co-existing malignancy diagnosis (two with breast cancer, one with melanoma, one with pancreatic cancer, one with renal cancer and one with cervical dysplasia) and 6 patients who had a diagnosis of a co-existing immune disorder (two with rheumatoid arthritis, two with ulcerative colitis, one with chronic urticaria with hypothyroidism and one with Sjogren’s syndrome). Nearly all malignancy cases occurred in older HAE patients (> 50 years) and malignancy was diagnosed before HAE in 3 of the patients.

**Conclusions:**

Our case series identified multiple hereditary angioedema (HAE) patients with co-existing malignancy and immune disorders. Based on these findings, we would advocate that physicians managing HAE patients should maintain a high index of suspicion for these conditions and that in patients with angioedema, C1 inhibitor deficiency and malignancy, a diagnosis of HAE should still be considered in addition to acquired angioedema (AAE).

## Background

Hereditary angioedema (HAE) is a rare autosomal dominant genetic condition with inherited deficiency or dysfunction of C1 inhibitor due to a mutation in the *SERPING1* gene. The overall prevalence of HAE is estimated to be about 1.1–1.6 per 100,000 [1]. C1 inhibitor is a key regulator protein of the classical and lectin complement pathways as well as the intrinsic coagulation and kinin generation pathways. Its deficiency is associated with increased kallikrein activity and subsequent bradykinin production leading to angioedema. C1 inhibitor deficiency can also lead to activation of the classical complement pathway leading to decreased C4 which is not directly involved in the development of angioedema [1]. Acquired angioedema (AAE) is a separate clinical entity caused by consumption of C1 inhibitor which can lead to a similar phenotype as HAE. However, unlike HAE, AAE is not an inherited disorder. AAE is associated with malignancies, particularly B cell lymphoproliferative diseases, but may also be caused by autoantibodies directed against the C1 inhibitor molecule [2]. In addition to the pharmacologic treatment of angioedema episodes as in HAE, treatment of the underlying malignancy or immune disorder associated with AAE can lead to decreased frequency of angioedema attacks [2]. In contrast to AAE, HAE is not known to be associated with either malignancy or immune disorders.

Malignancy is a prevalent condition with one study estimating 14.5 million Americans with a history of cancer were alive in 2014 [3]. A recent study involving mouse models suggested that complement may play a role in the anti-tumor response and they observed that complement 3a receptor (C3aR) deficiency or inhibition was protective against melanoma, breast and colon cancers [4]. Upon review of the literature however, we could not identify any published studies that have evaluated whether malignancy is more prevalent in the HAE population. Early identification of malignancy in at risk populations is critical to improve survival in cancer patients [5]. Conversely, immune disorders are relatively rare conditions in the general population with prevalence rates of individual autoimmune diseases (e.g., rheumatoid arthritis, ulcerative colitis) reported to be less than 1% [6, 7]. Both deficiencies and activation of complement have been associated with autoimmune diseases, in particular systemic lupus erythematosus (SLE) and vasculitis [8]. It is proposed that complement deficiencies may pre-dispose to autoimmunity due to defective clearance of immune complexes, impaired disposal of dying cells and development of abnormal peripheral tolerance [9]. A few studies have investigated whether there is an association between HAE and autoimmunity, however they have all had varying results [10–13]. However, a recent systemic review has identified 155 individual occurrences of HAE and autoimmune disease out of 2880 records, with SLE being the most common [14]. If malignancy and/or immune disorders are more prevalent in individuals with HAE, this could influence guidelines and screening of this patient population.

## Case presentation

This case series was conducted in a mixed pediatric/adult practice, private allergy clinic in Vancouver, British Columbia, Canada. Patient charts were reviewed if the patient had a confirmed diagnosis of HAE based on prior abnormally low C1 inhibitor functional testing on at least two separate occasions, and either confirmatory genetic testing showing a pathologic mutation in *SERPING1* or a family history of HAE. This diagnostic criterion of HAE is based on the International/Canadian Hereditary Angioedema Guidelines [15]. Individuals with other forms of angioedema such as acquired angioedema, ACE-inhibitor associated angioedema or hereditary angioedema with normal C1 inhibitor were excluded. Malignancy and immune disorder diagnoses were based on either patient reported history of condition or based on details provided in other medical subspecialty consultation letters (e.g., rheumatology, oncology). Malignancy included any form of malignant cells in any body system (including dysplasia). Immune disorders were defined as conditions involving dysfunction of the immune system including autoimmune diseases and primary immunodeficiencies.

49 charts of patients with HAE were reviewed and we identified six patients with a documented diagnosis of malignancy and six patients with a documented diagnosis of an immune disorder. A variety of malignant conditions were identified including two patients with breast cancer, one with melanoma, one with pancreatic cancer, one with renal cancer, and one with cervical dysplasia. All those with a diagnosis of malignancy were age 50 or greater at the time of diagnosis, with the exception of one patient who was diagnosed with cervical dysplasia in her early 40 s (Table 1). All individuals with a malignancy diagnosis either had a convincing family history of HAE following an autosomal dominant inheritance or had genetic testing that confirmed a diagnosis of HAE (as opposed to a suspected diagnosis of malignancy associated AAE). Three of these patients were diagnosed with malignancy prior to being diagnosed with HAE.

**Table 1:** Details of HAE patients with Malignancy Diagnosis

Patient number	Age at time of study	Sex	Age of HAE diagnosis	Details of HAE diagnosis	Type of malignancy	Age of malignancy diagnosis	Extent of malignancy and treatment
Patient 1	57	F	41	Multiple family members with HAE on paternal side	Breast	54	Invasive ductal carcinoma − ER/PR + , HER2 equivocal, treated with surgery and post-menopausal state
Patient 2	58	M	12	8 family members with HAE	Melanoma	56	Stage 3 with unknown primary, treated with surgery and chemotherapy
Patient 3	70	F	66	Sister, father and daughter with HAE	Pancreatic	70	Metastatic, passed away shortly after diagnosis
Patient 4	46	F	42	Mother and maternal aunt with HAE	Cervical dysplasia (ASC-H, prior HSIL)	Early 40 s, prior to HAE diagnosis	Localized, treated with Hysterectomy. Currently being investigated for bladder cancer
Patient 5	58	F	56	Brother and nephew with HAE	Breast	50	Treated with mastectomy and tamoxifen for five years
Patient 6	74	F	73	Mutation in *SERPING1*, suspected family history (currently being investigated)	Renal	62	Metastatic, treated with nephrectomy, immunotherapy, and localized radiation

*ER* estrogen receptor, *PR* progesterone receptor, *HER2* Herceptin receptor, *ASC-H* atypical squamous cells, *HSIL* high-grade squamous intraepithelial lesion

Six patients reported a diagnosis of an immune disorder including two patients with ulcerative colitis, two patients with rheumatoid arthritis, one patient with Sjogren’s syndrome, and one patient with hypothyroidism and chronic spontaneous urticaria (CSU). The youngest patient identified with an immune disorder was Patient 8, who indicated that she was diagnosed with ulcerative colitis at age 19 (Table 2). The age of diagnosis of the immune disorder was not available for any of the other patients, however at the time of our data collection, three patients were aged 18–49 and three patients were aged 50 or older. Both patients with ulcerative colitis (Patient 8 and Patient 11), required management with biologic therapy. No patients were identified with a primary immunodeficiency.

**Table 2:** Details of HAE patients with immune disorder diagnosis

Patient number	Age at time of study	Sex	Immune disorder	Other details and treatment
Patient 7	41	F	Rheumatoid arthritis	Managed with hydroxychloroquine and methotrexate
Patient 8	31	F	Ulcerative colitis	Managed with infliximab
Patient 9	90	F	Sjogren’s syndrome	
Patient 10	42	F	Hypothyroidism and CSU	
Patient 11	64	M	Ulcerative colitis	Managed with ustekinumab
Patient 12	67	M	Rheumatoid arthritis	Also, history of renal transplant due to ESRD secondary to type 2 DM, immunosuppressed with MMF, tacrolimus and prednisone

*CSU* chronic spontaneous urticaria, *ESRD* end-stage renal disease, *DM* diabetes mellitus, *MMF* mycophenolate mofetil

## Discussion and conclusions

Nearly all of the cases of malignancy we identified in our HAE population occurred in older adults (> 50 years) and we detected a variety of malignant conditions including breast cancer, melanoma, pancreatic cancer, renal cancer and cervical dysplasia. These observations raise the possibility of an increased incidence of malignant conditions in older HAE patients (> 50 years). There has not been any previous published data that has investigated if patients with HAE are at an increased risk of malignancy. In our study, three patients were formally diagnosed with HAE after their malignancy was diagnosed. As HAE and AAE can present very similarly, we would advocate that older patients with malignancy who develop C1 inhibitor deficiency and angioedema, a high index of suspicion of HAE should be maintained as this could potentially identify relatives with undiagnosed HAE. Further cross-sectional studies with a larger sample size would be needed to determine if there is truly an associated relationship.

We identified six HAE patients with a diagnosis of a co-existing immune disorder. Our data is consistent with other studies that have showed a higher incidence of autoimmune conditions in patients with HAE [8]. The exact pathophysiology to explain this association is yet to be elucidated, although many complement deficiencies are known to be associated with autoimmunity. Inherited C1q, C1r, C1s, C4 and C2 deficiency have all been found to be associated with an increased incidence of SLE [8]. A small retrospective study observed that HAE patients receiving C1 inhibitor replacement therapy had subsequent fewer physician visits for autoimmune disorders than patients with HAE who were not treated with C1 inhibitor replacement and concluded that normalization of complement levels may have a positive impact on coexisting autoimmune diseases [16]. These findings have yet to be verified on further studies, but support the hypothesis of complement deficiencies contributing to autoimmunity.

Our case series has identified multiple hereditary angioedema (HAE) patients with co-existing malignancy and immune disorders. Although we are limited by our small sample size and lack of a control population, based on these findings we would recommend that physicians managing HAE patients should maintain a high index of suspicion of co-existing malignancy and immune disorders. We would also recommend that a diagnosis of HAE should still be considered in patients presenting with angioedema, C1 inhibitor deficiency and malignancy in addition to AAE.

## Data Availability

All data generated or analyzed during this study are included in this published article.

## Abbreviations

HAE: Hereditary angioedema
AAE: Acquired angioedema
CSU: Chronic spontaneous urticaria
